# Trajectories in Glycemic Control over Time Are Associated with Cognitive Performance in Elderly Subjects with Type 2 Diabetes

**DOI:** 10.1371/journal.pone.0097384

**Published:** 2014-06-02

**Authors:** Ramit Ravona-Springer, Anthony Heymann, James Schmeidler, Erin Moshier, James Godbold, Mary Sano, Derek Leroith, Sterling Johnson, Rachel Preiss, Keren Koifman, Hadas Hoffman, Jeremy M. Silverman, Michal Schnaider Beeri

**Affiliations:** 1 Memory clinic, Sheba Medical Center, Tel Hashomer, Ramat Gan, Israel; 2 Department of Family Medicine, University of Tel Aviv, Tel Aviv, Israel; 3 Department of Psychiatry, Icahn School of Medicine at Mount Sinai, New York, United States of America; 4 Department of Preventive Medicine, Icahn School of Medicine at Mount Sinai, New York, United States of America; 5 Department of Medicine, Icahn School of Medicine at Mount Sinai, New York, United States of America; 6 Geriatric Research Education and Clinical Center, Madison VA Hospital and Alzheimer’s Disease Research Center, Department of Medicine, University of Wisconsin, WI, United States of America; 7 Maccabi Health Services, Tel Aviv, Israel; 8 The Joseph Sagol Neuroscience Center, Sheba Medical Center, Ramat Gan, Israel; Nathan Kline Institute and New York University School of Medicine, United States of America

## Abstract

**Objective:**

To study the relationships of long-term trajectories of glycemic control with cognitive performance in cognitively normal elderly with type 2 diabetes (T2D).

**Methods:**

Subjects (n = 835) pertain to a diabetes registry (DR) established in 1998 with an average of 18 HbA1c measurements per subject, permitting identification of distinctive trajectory groups of HbA1c and examining their association with cognitive function in five domains: episodic memory, semantic categorization, attention/working memory, executive function, and overall cognition. Analyses of covariance compared cognitive function among the trajectory groups adjusting for sociodemographic, cardiovascular, diabetes-related covariates and depression.

**Results:**

Subjects averaged 72.8 years of age. Six trajectories of HbA1c were identified, characterized by HbA1c level at entry into the DR (Higher/Lower), and trend over time (Stable/Decreasing/Increasing). Both groups with a trajectory of decreasing HbA1c levels had high HbA1c levels at entry into the DR (9.2%, 10.7%), and high, though decreasing, HbA1c levels over time. They had the worst cognitive performance, particularly in overall cognition (p<0.02) and semantic categorization (p<0.01), followed by that of subjects whose HbA1c at entry into the DR was relatively high (7.2%, 7.8%) and increased over time. Subjects with stable HbA1c over time had the lowest HbA1c levels at entry (6.0%, 6.8%) and performed best in cognitive tests.

**Conclusion:**

Glycemic control trajectories, which better reflect chronicity of T2D than a single HbA1c measurement, predict cognitive performance. A trajectory of stable HbA1c levels over time is associated with better cognitive function.

## Introduction

Type 2 diabetes (T2D) is one of the most common chronic diseases worldwide and is associated with an increased risk for cognitive decline and dementia [1], [2]. Chronic hyperglycemia and alterations of cellular homeostasis, characteristic of T2D, lead to diffuse vascular damage and multi-organ dysfunction [3]. Hyperglycemia is a key determinant of both macrovascular (e.g. myocardial infarction, stroke) and microvascular (e.g. retinopathy) complications of T2D, and there is extensive evidence showing that both acute and chronic hyperglycemia are deleterious [4], [5], [6]. Hemoglobin A1c (HbA1c) levels (the gold standard measurement of glycemic control), even in non-diabetic individuals [7], are associated with cognitive performance [8], [9] and brain volume [10], [11]. Based on the beneficial effect of good glycemic control in preventing other diabetes complications [12], it is clinically reasonable to strive for an optimal level of glycemic control [13] in order to mitigate or perhaps prevent cognitive decline and dementia [13]. Good glycemic control has been demonstrated by some [14], [15] to be associated with better cognitive function even in non-T2D individuals [16]. However, strict glycemic control achieved by anti-diabetic medications has been shown to increase risk for morbidity and mortality in some T2D subjects [17] and therefore cannot be homogenously applied. It is therefore relevant and useful as an initial approach to study the association of trajectories in glycemic control over time–reflecting long-term T2D processes rather than glycemic control at a certain period in time, with cognition. Such an approach may form a basis for identification of T2D subjects in which achievement of good glycemic control may be safe and efficacious as a means for dementia prevention.

Studies on the relationship of other cardiovascular risk factors (e.g blood pressure and weight) and dementia have demonstrated that trends over time–not only mean levels–were associated with increased risk for dementia [18], [19], [20]. Trends in glycemic control among T2D subjects, as reflected in trajectories of repeated HbA1c measurements over years, were associated with mortality [21]. However, to the best of our knowledge, the relationship of such trajectories with cognitive function has not been studied. The present study examined the relationship of empirically developed trajectories of HbA1c levels over time and cognitive function in a cognitively normal cohort of elderly T2D subjects participating in the Israel Diabetes and Cognitive Decline (IDCD) study, a longitudinal investigation of the relationship of long-term T2D characteristics with cognitive decline.

## Results

### Description of the Sample

There were 1288 subjects who passed the preliminary screening, expressed interest in participating, were approached by a study physician and signed informed consent. Of them, 282 (21.1%) were excluded from the study due to incompatibility with eligibility criteria (based on physician assessment) and 109 (8.5%) refused to continue their participation in the study, so 897 subjects remained active participants. The study consists of 835 subjects who had complete data on sociodemographic, cardiovascular, and diabetes-related covariates as well as GDS score and had at least 2 HbA1c measurements. Table 1 describes the sample characteristics and compares the HbA1c trend categories using analysis of variance for continuous outcomes, and Pearson’s chi square for dichotomous outcomes. The mean age at entry into the IDCD was 72.75 (4.63) years, with a majority of males (60%). The average levels of total, HDL, and LDL cholesterol, diastolic blood pressure, and GFR were consistent with the general elderly population in this age range. Systolic blood pressure was higher than in the general population but similar to that observed in other T2D samples [22]. Most subjects were treated with oral anti-diabetic medications (79%), 1% were treated with insulin only, 9% were treated with a combined therapy of oral anti-diabetic medications and insulin and 11% were controlled by diet only. The mean number of HbA1c measurements per subject was 17.9 (SD = 9.6). Mean duration of inclusion in the DR was 8.70 (2.64) years, mean HbA1c levels at entry into the DR and at follow up were 6.95% (1.32) and 6.96% (1.01) respectively.

**Table 1:** Characteristics of the sample by HbA1c* Trajectory group.

Variable	Lower Stable*	Higher Stable*	Lower Increasing	Higher Increasing	Lower Decreasing	Higher Decreasing	Total	p-value
	N = 227	N = 365	N = 123	N = 46	N = 59	N = 15	N = 835	
**Years in Diabetes Registry**	8.35 (2.58)	8.35 (2.81)	9.36 (2.32)	8.88 (2.57)	10.13 (1.7)	10.95 (0.56)	8.70 (2.64)	**<0.0001**
**HbA1c at entry into Diabetes Registry**	5.96 (0.67)	6.84 (0.94)	7.26 (0.84)	7.76 (0.95)	9.19 (1.4)	10.73 (0.97)	6.95 (1.32)	**<0.0001**
**Mean HbA1c During Follow-Up**	6.01 (0.28)	6.70 (0.23)	7.45 (0.23)	8.35 (0.34)	7.61 (0.36)	9.22 (0.45)	6.82 (0.77)	**<0.0001**
**Current** **age**	72.99 (4.75)	72.91 (4.66)	72.52 (4.46)	70.78 (4.16)	73.63 (4.48)	69.73 (3.35)	72.75 (4.63)	**0.0027**
**Education (years)**	13.43 (3.47)	13.21 (3.71)	13.12 (3.18)	12.59 (2.8)	12.75 (2.71)	12.00 (2.80)	13.17 (3.45)	0.3857
**HDL** *	48.39 (11.24)	48.06(10.68)	47.93 (11.58)	43.97 (8.35)	46.73 (9.44)	44.98 (11.8)	47.76 (10.82)	0.1427
**LDL** *	102.15 (19.12)	103.69 (19.82)	98.38 (19.22)	97.84 (27.38)	95.56 (14.46)	93.89 (22.7)	101.42 (19.9)	**0.0050**
**Total cholesterol**	179.65 (24.69)	182.69 (25.96)	180.14 (23.61)	182.28 (28.45)	172.31 (18.34)	167.58 (27.52)	180.46 (25.12)	**0.0184**
**Systolic** **BP** *	133.4 (8.86)	134.88(9.11)	135.45 (9.42)	137.70 (9.98)	135.38 (12.04)	133.99 (8.01)	134.74 (9.39)	0.0659
**Diastolic** **BP**	76.85 (4.74)	77.40(4.81)	76.90 (4.52)	77.52 (4.66)	75.15 (5.84)	76.18 (5.54)	77.00 (4.85)	**0.0319**
**GFR** *	80.38 (23.87)	81.71(27.13)	79.33 (25.98)	83.31 (32.5)	77.82 (25.73)	91.43 (24.42)	80.99 (26.29)	0.4852
**GDS** *	1.00 [0–9]	1.00 [0–11]	2.00 [0–9]	2.00 [0–10]	1.00 [0–14]	1.00 [0–9]	1.00 [0–14]	0.3249
**Diabetes medication group**								
***Oral antidiabetic Only***	164 (72%)	324 (89%)	103 (84%)	25 (54%)	45 (76%)	2 (13%)	663 (79%)	**<0.0001**
***Insulin Only***	3 (1%)	2 (1%)	2 (2%)	1 (2%)	1 (2%)	0 (0%)	9 (1%)	
***Insulin+Oral antidiabetic***	3 (1%)	6 (2%)	17 (14%)	20 (43%)	13 (22%)	12 (80%)	71 (9%)	
***None***	57 (25%)	33 (9%)	1 (1%)	0 (0%)	0 (0%)	1 (7%)	92 (11%)	

*HbA1c = hemoglobin A1c, HDL = High density lipoprotein, LDL = low density lipoprotein, GFR = glomerular filtration rate, GDS = geriatric depression scale.

**Figure 1:**
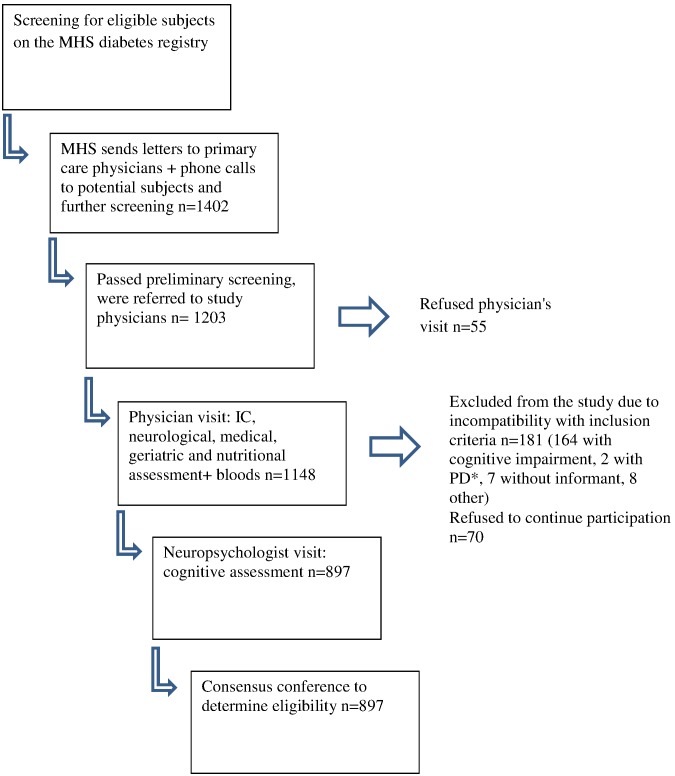
Trajectories in HbA1c levels. Groups: 1 = lower Stable, 2 = Higher Stable, 3 = Lower Increasing, 4 = Higher Increasing, 5 = Lower Decreasing, 6 = Higher Decreasing.

The PROC TRAJ analysis identified six trajectories, with each characterized by (1) the HbA1c level at entry into the DR (intercept), and (2) the trend over time in the registry (figure 1). Three types of trends (stable, increasing and decreasing) in HbA1c over time were observed, and within each type there were two groups with nearly parallel trends but with different intercepts (HbA1c levels at entry to the Registry). For convenience purposes, the groups are referred to by (1) whether it has a Higher or Lower intercept within each type of trend (the terms higher and lower were used for descriptive purposes rather than for clinical purposes, in order to differentiate, within each trend in HbA1c, those who entered with a relatively higher from those who entered with a relatively lower HbA1c into the DR), and (2) the type of trend in HbA1c over time, either Stable, Increasing or Decreasing. Groups differed in number of years in the DR (longest duration for the groups whose HbA1c decreased, followed by the groups whose HbA1c increased and finally by the groups that remained stable); HbA1c levels at baseline was highest for the groups whose HbA1c decreased, followed by the groups whose HbA1c increased and lowest for the groups that remained stable; mean HbA1c during follow up was highest for the Higher Decreasing and Higher Increasing groups and lowest for the two groups that remained stable. Differences were found for anti-diabetic medications use such that the Higher Decreasing group had the lowest use of oral anti-diabetics only and the highest use of both oral anti-diabetics and insulin. Additional differences were observed for age at entry into the IDCD, LDL cholesterol, and diastolic blood pressure.

### The Association of HbA1c Trajectories with Cognitive Function

The overall models were significant for overall cognition, semantic categorization, and executive functions (see Table 2). For descriptive purposes, Table 3 presents comparisons among the different trajectories. Mean overall cognitive z-score was significantly lower in the Higher Decreasing group compared to all other groups except Higher Increasing- in which the difference approached significance. Similarly, for semantic categorization, mean z-scores were lower for the Higher Decreasing group compared to all other groups (approaching significance for the Lower Decreasing group). The Lower Decreasing group had poorer z-scores than the other groups except for the Higher Increasing group. Scores for executive function were lower for Higher Decreasing compared to all other groups except the Higher Increasing group which by itself had poorer function compared to the two stable groups. The mean z-score for the Lower Increasing group was significantly lower than that of the Lower Stable group and approached significance compared to the Higher Stable group (tables 2 and 3). Finally, no significant differences were observed among groups in the episodic memory and attention/working memory domains.

**Table 2:** Cognitive scores (Z scores) in groups of subjects defined by HbA1c at entry into the Diabetes Registry and trend over time in the registry*.

Group	Z score overall cognitive function	Standard Error	Pr>|t|	Overall P-value
Overall cognitive score				
Lower Stable (n = 232)	1.39	0.79	0.0789	
Higher Stable (n = 371)	1.13	0.76	0.1366	
Lower Increasing (n = 124)	0.72	0.88	0.4142	
Higher Increasing (n = 47)	−0.76	1.20	0.5273	0.0479
Lower Decreasing (n = 61)	−0.03	1.11	0.9765	
Higher Decreasing (n = 15)	−4.93	2.00	0.0140	
Episodic memory				
Lower Stable (n = 232)	0.16	0.24	0.4943	
Higher Stable (n = 371)	0.25	0.23	0.2674	
Lower Increasing (n = 124)	0.16	0.26	0.5511	
Higher Increasing (n = 47)	−0.59	0.36	0.1032	0.3275
Lower Decreasing (n = 61)	0.25	0.33	0.4422	
Higher Decreasing (n = 15)	−0.13	0.60	0.8304	
Semantic categorization				
Lower Stable (n = 232)	0.16	0.24	0.4875	
Higher Stable (n = 371)	0.10	0.23	0.6547	
Lower Increasing (n = 124)	0.19	0.27	0.4803	
Higher Increasing (n = 47)	−0.02	0.36	0.9535	0.0252
Lower Decreasing (n = 61)	−0.52	0.33	0.1152	
Higher Decreasing (n = 15)	−1.63	0.60	0.0066	
Attention/working memory				
Lower Stable (n = 232)	0.04	0.23	0.8551	
Higher Stable (n = 371)	−0.18	0.22	0.3948	
Lower Increasing (n = 124)	0.05	0.25	0.8573	
Higher Increasing (n = 47)	0.09	0.34	0.8004	0.2848
Lower Decreasing (n = 61)	−0.09	0.32	0.7766	
Higher Decreasing (n = 15)	−1.10	0.57	0.0568	
Executive function				
Lower Stable (n = 232)	0.88	0.29	0.0021	0.0069
Higher Stable (n = 371)	0.62	0.27	0.0241	
Lower Increasing (n = 124)	0.12	0.32	0.7137	
Higher Increasing (n = 47)	−0.26	0.44	0.5498	
Lower Decreasing (n = 61)	0.33	0.40	0.4130	
Higher Decreasing (n = 15)	−1.48	0.73	0.0418	

*Analysis of covariance to estimate and compare mean z-scores in the different cognitive domains among the trajectory groups, adjusting for sociodemographic, cardiovascular, diabetes-related covariates (years in the DR and anti-diabetic medications), and GDS score.

**Table 3:** Comparisons between cognitive scores in groups of subjects defined by HbA1c at entry into the Diabetes Registry and trend over time in the registry.

Overall cognitive score						
	Lower Stable	Higher Stable	Lower Increasing	Higher Increasing	Lower Decreasing	Higher Decreasing
**Lower Stable**		0.6680	0.4191	0.0852	0.1845	**0.0021**
**Higher Stable**			0.5890	0.1143	0.2543	**0.0029**
**Lower Increasing**				0.2374	0.4977	**0.0059**
**Higher Increasing**					0.6075	0.0508
**Lower Decreasing**						**0.0213**
**Episodic memory**						
	**Lower Stable**	**Higher Stable**	**Lower Increasing**	**Higher Increasing**	**Lower Decreasing**	**Higher Decreasing**
**Lower Stable**		0.6207	0.9893	0.0451	0.7692	0.6373
**Higher Stable**			0.6768	0.0192	0.9895	0.5327
**Lower Increasing**				0.0476	0.7708	0.6405
**Higher Increasing**					0.0473	0.4729
**Lower Decreasing**						0.5465
**Semantic categorization**						
	**Lower Stable**	**Higher Stable**	**Lower Increasing**	**Higher Increasing**	**Lower Decreasing**	**Higher Decreasing**
**Lower Stable**		0.7303	0.9248	0.6202	**0.0320**	**0.0036**
**Higher Stable**			0.7011	0.7324	**0.0403**	**0.0045**
**Lower Increasing**				0.5798	**0.0334**	**0.0031**
**Higher Increasing**					0.2363	**0.0118**
**Lower Decreasing**						0.0817
**Attention/working memory**						
	**Lower Stable**	**Higher Stable**	**Lower Increasing**	**Higher Increasing**	**Lower Decreasing**	**Higher Decreasing**
**Lower Stable**		0.1944	0.9853	0.8978	0.6678	0.0538
**Higher Stable**			0.2826	0.4274	0.7462	0.1184
**Lower Increasing**				0.9080	0.6707	0.0524
**Higher Increasing**					0.6622	0.0536
**Lower Decreasing**						0.0994
**Executive function**						
	**Lower Stable**	**Higher Stable**	**Lower Increasing**	**Higher Increasing**	**Lower Decreasing**	**Higher Decreasing**
**Lower Stable**		0.2306	**0.0103**	**0.0115**	0.1526	**0.0015**
**Higher Stable**			0.0642	**0.0422**	0.4305	**0.0045**
**Lower Increasing**				0.4054	0.6008	**0.0317**
**Higher Increasing**					0.2508	0.1153
**Lower Decreasing**						**0.0190**

After applying the Bonferroni-Holms step down correction to account for multiple comparisons, the following pairwise comparisons remained significant: for overall cognition, the comparison of the Higher Decreasing group to the Lower stable group (p = 0.0021) and to the Higher Stable group (p = 0.0029), for semantic categorization, the comparison of the Higher Decreasing group to the Lower Increasing group (p = 0.0031) and for executive function, the comparison of the Higher Decreasing group with the Lower Stable group (0.0015).

A secondary analysis demonstrated that higher mean HbA1c was associated with lower scores in overall cognitive score (p = 0.01) and executive functions (p = 0.0003) and with a trend for lower scores in semantic categorization (p = 0.06). Higher standard deviation in HbA1c was associated with lower scores in executive function (p = 0.025) and a trend towards lower scores in overall cognitive score (p = 0.07).

Exclusion of the handful of cases in which there was a gap between the CDR score and the MMSE scores did not affect the results.

## Discussion

The present study demonstrated that among elderly T2D subjects, the trajectories of glycemic control over time were associated with cognitive functioning in the cognitive domains of semantic categorization, executive function and overall cognition. Subjects with a trajectory of decreasing HbA1c levels over the years, were characterized by very high (mean = 10.73%) or high (mean 9.19%) HbA1c levels at entry into the DR, and high, though decreasing, HbA1c levels over their T2D course. These subjects had the poorest cognitive performance. Their performance was followed by that of subjects whose HbA1c at entry into the DR was relatively high (mean = 7.76) and increased over time. Subjects with stable HbA1c throughout the years, had the lowest HbA1c levels at all times and performed best in cognitive tests. These analyses were adjusted for sociodemographic, cardiovascular, and T2D-related variables. Trajectories in HbA1c over time were not associated with episodic memory or attention/working memory. Importantly, the trajectories were not defined a-priori and were not based on clinical cutoffs but were rather empirical. Following correction of the analysis for multiple comparisons, the comparison between the most extreme trajectories remained significant in overall cognition, semantic categorization and executive functions. Examining trajectories in HbA1c as predictors of T2D outcomes is advantageous since they describe better the natural history of T2D, with varying degrees of glycemic control over time [23]. In contrast to variability around the mean, the trajectories capture the true course of T2D through a combination of its inherent components (baseline HbA1c, overall slope of the trajectory, the mean, the end levels etc), rather than each component separately, thus allowing detection of subgroups of change in HbA1c that follow a distinct course.

To the best of our knowledge, this is the first study to investigate the association of trajectories in glycemic control over time with cognitive functioning. Most studies reporting on the association of T2D and glycemic control with cognitive outcomes (or cognitive related outcomes such as brain volume), used diagnosis of T2D or degree of glycemic control at entry as predictors, as opposed to glycemic control over time.

The decrease in HbA1c levels over time in subjects with very high HbA1c at baseline, suggests that these subjects were treated with anti-diabetes medications as clinically warranted. Indeed, they had the highest percentage of use of both hypoglycemic medications and insulin. Nevertheless, these subjects failed to reach the clinically acceptable goals of HbA1c despite treatment, and were thus at higher risk for the detrimental effects of chronic hyperglycemia on cognition [24]. The trajectories observed suggest that these subjects suffer from a more “aggressive” course of T2D, possibly underlying the poorer cognitive functioning in this group. Alternatively, the anti-diabetic treatments may have exposed these subjects to an increased risk for hypoglycemic episodes [25] and to the implications of the latter on cognition [26]. The analysis was adjusted for sociodemographic (age, sex, and years of education), cardiovascular (glomerular filtration rate calculated by the MDRD formula, total cholesterol, HDL, LDL, diastolic and systolic blood pressure), T2D related factors (estimated duration of T2D and diabetes medications) and depression (based on GDS score). Nevertheless, we cannot rule out the possibility that an overall higher severity of T2D in the two groups with decreasing HbA1c over time, contributed to their poorer cognitive function.

It is important to note the differences between the two groups with a trajectory of decrease in HbA1c over time; both had high HbA1c levels throughout their follow up in the DR, however, the group with the lower levels (Lower Decreasing) performed better in overall cognition and executive function than the group with the highest levels (Higher Decreasing). These differences suggest that cognitive function in T2D may be better preserved when aiming towards lower HbA1c levels, even without achieving optimal glycemic control. Trajectories in HbA1c levels over time were associated with cognitive decline even in non-diabetic, non-demented elderly subjects [16] suggesting that long-term peripheral glucose levels per se, not only in the context of T2D, may be associated with biological mechanisms for neuronal dysfunction/neurodegeneration and subsequent cognitive compromise. This hypothesis is further supported by studies showing a negative association between HbA1c levels (within the normal range in non-demented non-diabetic individuals) and brain volume at 6 years follow up [10].

A trajectory of increasing HbA1c levels over time has previously been demonstrated to be associated with increased mortality in a dose-response manner in a cohort of 8,812 T2D subjects, with a mean follow up duration of 4.5 years [21]. Consistent with that, the present results show that the groups with increasing HbA1c over time had poorer cognitive performance compared to subjects with stable HbA1c levels over time.

The groups that performed best on cognition were those with stable and relatively low HbA1c levels over the course of the disease. Findings regarding the role of glycemic control in prevention of other T2D complications are heterogeneous, with results varying by study population, outcome and type of intervention [27]. In the ACCORD study, higher levels of HbA1c at entry were associated with lower cognitive performance (based on the Digit Symbol Substitution Test-DSST) at <45 days afterwards [8]. Interestingly, in the 40-months follow up phase of the ACCORD study, the intensive treatment arm (aiming for HbA1c less than 6.0%) was associated with greater total brain volume but not with DSST score, compared to standard treatment (aiming for HbA1c between 7% and 7.9%) [11]. The authors concluded that such results, combined with the increased mortality in the intensive care group and the non-significant effects on other ACCORD outcomes, do not support the use of intensive therapy to reduce the adverse effects of T2D on the brain. A departure from some disease-state homeostasis by enforcing too strict glycemic control was hypothesized to render some subjects to hypoglycemic episodes or other conditions with negative consequences on cognition. The present results suggest that stabilization of glycemic control over many years may be advantageous.

Despite lower cognitive function in some domains, the subjects participating in the IDCD were all broadly within cognitive normal limits. Previous studies have demonstrated that people with normal, albeit lower range of cognitive function, are at higher risk of developing cognitive decline and dementia [28]. The numerous HbA1c assessments available for the IDCD cohort, enabled detection of trajectories in glycemic control that are particularly deleterious, suggesting target T2D subjects who are at higher risk for lower cognitive function and for future incident dementia and thus candidates for evolving therapies to maintain or slow cognitive decline.

The HbA1c trends were measured from several years before the cognitive assessment, and the cognitive outcomes were all within the normal range, suggesting that glycemic control affects cognition rather than an incipient dementing process affecting glycemic control. However, this study is observational and at this point only cross-sectional cognitive data is available. Thus causality should not be inferred; we cannot rule out the possibility that poor, albeit normal, cognitive performance is associated with poor self-care, leading to high HbA1c levels. When longitudinal cognitive follow-ups become available, evaluations of the relationships of patterns of glycemic control with cognitive decline, and incident MCI and dementia will elucidate the direction of the relationship between glycemic control and cognition. Examination of the association, within each trajectory group, of each trajectory component (the mean HbA1c, standard deviation in HbA1c and change in HbA1c), to assess their unique contribution to cognition was not possible. Such an examination would require at least three different slopes (trend patterns) at each different baseline HbA1c, along with at least 2 different standard deviations (e.g. lower and higher) around each of the slopes at each baseline HbA1c. This would permit a variety of scenarios such as high baseline HbA1c, negative slope, low standard deviation, etc. However, the trajectories that we found are empirical, reflecting the true reality of our sample, and converged to 6 trajectories, which do not cover all the spectrum of trajectories to, theoretically, enable such an examination. This is a limitation of an observational study, but, a randomized trial where patients with high initial HbA1c levels were treated/not treated to decrease it, would be unethical. We nevertheless, performed, on the full sample, secondary analyses examining the relationships of mean and standard deviation of HbA1c with the cognitive outcomes and found that higher mean and standard deviation in HbA1c measurements over time were associated with lower scores in overall cognitive score and in executive functions, consistent with the trajectories results. Brain imaging was not performed in this study, thus limiting our ability to evaluate the contribution of cerebrovascular abnormalities to the association of trends in glycemic control with cognition. This is particularly relevant both because T2D is a vascular disease, but also because trajectories of HbA1c were not associated with episodic memory, suggesting non AD-related mechanisms. Entry into the DR, rather than time of T2D diagnosis, which was not available to us, is referred to as “baseline”. Although women are slightly under-represented in the study, sex was one of the covariates in the comparison of groups. Israel has a strong family oriented culture, so a major role in grand- parenting was the primary reason of refusal by women to participate in the study.

Additional strengths of this study include the large sample, validated T2D diagnosis for each subject, an average of 18 HbA1c measurements permitting investigation of trajectories of HbA1c over time, strong validity for risk factor levels and medical diagnosis, and a thorough cognitive evaluation.

## Methods

### Ethics Statement

The study was approved by the IRB committees of the Sheba Medical Center, Israel, the Maccabi Health Services (MHS), Israel and the Icahn School of Medicine at Mount Sinai, NY. Informed consent was signed by all study participants.

### Population

This study consisted of elderly (≥65 years old) T2D subjects in the IDCD, a collaboration between the Icahn School of Medicine at Mount Sinai, NY, the Sheba Medical Center, Israel, and the Maccabi Health Services (MHS), Israel. Detailed methods have been presented [29]. IDCD subjects were randomly selected from the approximately 11,000 T2D individuals aged ≥65 years that are in the Diabetes Registry (DR) of the MHS, the second largest HMO in Israel. The MHS DR is an integral part of the MHS Electronic Patient Record system, which was established in 1998 to facilitate disease management and to improve treatment. The DR has collected detailed laboratory, medication, and diagnoses information since 1998 [30]. The present analysis assesses the relationship of long term trajectories in HbA1c, since the subject’s entry into the MHS DR diabetes registry (the earliest time was 1998, when the diabetes registry was established) until the IDCD baseline cognitive evaluation (2010–2011).

Any of the following criteria should be met in order to be included in the MHS DR: 1) HbA1c >7.25%, 2) Glucose >200 mg/dl on two exams more than three months apart, 3) purchase of anti-diabetic medication twice within three months supported by a HbA1c >6.5% or Glucose >125 mg/dl within half a year, 4) diagnosis of T2D (ICD9 code) by a general practitioner, internist, endocrinologist, ophthalmologist, or T2D advisor, supported by a HbA1c >6.5% or Glucose >125 mg/dl within half a year. These criteria have been validated by twenty physicians in MHS against their own practice records [30]. Additionally, age specific prevalence rates were similar to those of a DR of another large HMO in Israel^28^.

### Eligibility Criteria for the IDCD

(1) In the MHS DR, i.e. diagnosed with T2D (2) lives in the central area of Israel (near Tel Aviv), (3) 65 years of age or above, (4) cognitively normal (not suffering from dementia or MCI) at entry into the IDCD (based on a multidisciplinary consensus conference), (5) two or more HbA1c measurements in the DR, (6) has an informant, and (7) speaks Hebrew fluently. Potential subjects were excluded if they had an ICD code for dementia or its subtypes, treatment with prescribed cholinesterase inhibitors, or had a major psychiatric or neurological condition (such as schizophrenia, stroke or Parkinson’s disease) that could affect cognitive performance.

### IDCD Subjects’ Recruitment Process (Figure 2)

An algorithm including the eligibility criteria that were available in the MHS Electronic Patient Record was used to randomly select the subjects. After random selection of subjects, letters were sent by MHS to the primary care physicians, asking for permission to contact each patient regarding the study. If the doctor agreed, a letter was sent to the patient briefly describing the study and saying he or she would be contacted by phone in the following two weeks. The study coordinator then called the patient and invited participation in the study, after determining fluency in Hebrew and that there was an informant willing to provide information about the subject’s health. The social structure of Israel is such that most elderly individuals live with or near their extended family, so few are excluded for this reason. A second informant was sought to be a replacement if necessary. Subjects were assessed in two phases, typically at their residence, or at the Sheba Medical Center memory clinic, according to their preference. In the first meeting, a study physician obtained signed informed consent; performed medical, neurological, geriatric and nutritional (Food Frequency Questionnaire- FFQ) assessments; and drew blood for inflammatory markers (Il-6, CRP), and haptoglobin and ApoE genotypes. The second meeting, conducted within two weeks after the physician’s assessment, involved a neuropsychologist administering to the subject a cognitive battery, and both to the subject and an informant questionnaires for cognitive and functional impairment, and for depression and behavioral disturbances characteristic of dementia.

**Figure 2:**
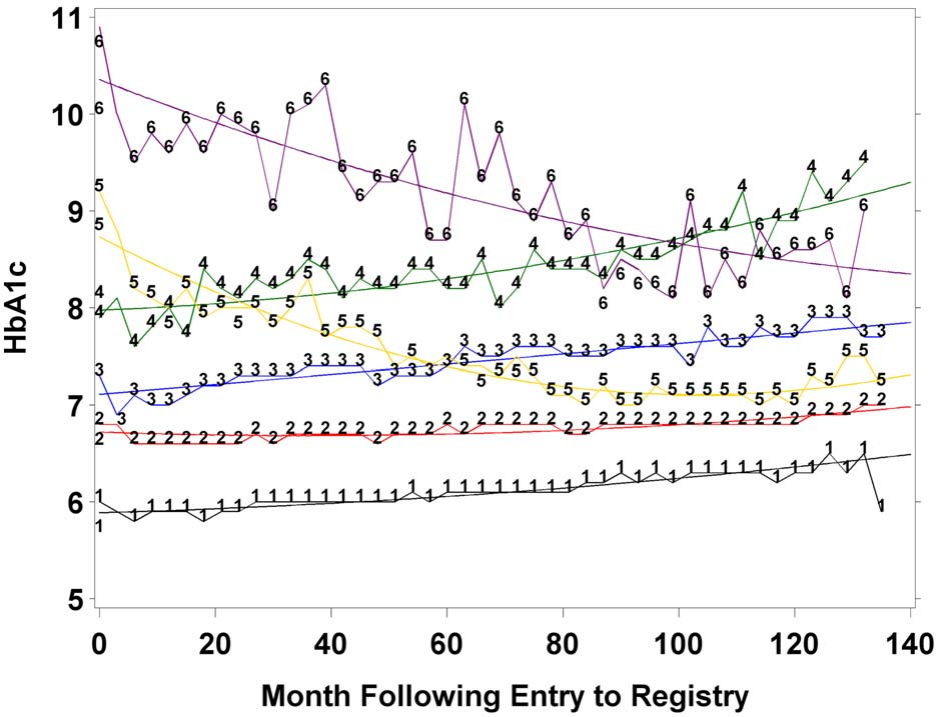
Study flow chart.

### Cognitive Assessment

The present analysis examined the association between long term trends in glycemic control (using all HbA1c measurements present in the subjects’ DR from their entry into the registry until IDCD initiation) with cognitive outcomes based on cognitive assessment that was performed at entry into the IDCD (baseline cognitive assessment). The cognitive assessment includes the scales and neuropsychological tests described below and takes approximately 2 hours to be administered. The study is ongoing and follow-up cognitive assessments have recently begun and do not enable, at this phase, report of the association of trajectories of glycemic control and changes in cognition over time.

#### Clinical Dementia Rating (CDR) scale

This scale assesses, through an interview with the subject and an informant, the severity of cognitive and functional impairment in 6 domains: memory, orientation, judgment and problem solving, community affairs, home and hobbies and personal care. A score of 0 represents normal cognition (an inclusion criteria for the IDCD study), 0.5 represents questionable dementia, and scores of 1 through 3 reflect increasing severity of dementia [31], [32].

#### Mini Mental State Exam (MMSE)

This questionnaire assesses orientation, concentration, memory, praxis and language [33]. Maximal score is 30.

#### Geriatric Depression Scale (GDS)

This is a self-report scale designed to be simple to administer and not to require the skills of a trained interviewer. The original instrument is a 30-item questionnaire developed for the assessment of depressive symptoms in older people [34]. The answers have a yes/no format. In the present study, the short version of the scale (composed of 15 items), was administered [35].

#### Neuropsychological battery

A thorough neuropsychological battery that characterizes the breadth of cognitive functions is administered. The battery is administered by experienced and certified interviewers which are blind to the diabetes related data. The neuropsychological evaluation is the basis for the outcome measures described below and includes the following tests:

1. Word List Memory [36]- This is a free recall memory test that assesses learning ability for new verbal information.
2. Similarities. This is a subtest from the Wechsler Adult Intelligence Scale-Revised. The test measures abstract thinking by asking the subject to state how pairs of words (e.g., egg/seed) are alike [37].
3. Letter fluency [38]: In three one-minute trials, this test of phonemic fluency assesses the ability to name as many words as possible beginning with three Hebrew letters-beit, gimel, shin [39].
4. Digit Span [40]: This is a subtest of the WAIS-III. The Digit Forward section assesses attention by reading sequences of digits to the subject for immediate verbatim repetition. Then the Digit Backwards section consists of sequences to be repeated in reverse order.
5. Diamond Cancellation Test: This test is used to assess vigilance and speeded attention. It requires subjects to identify target stimuli (diamonds) randomly interspersed among distractor stimuli on a sheet of 8.5-by-11 paper.
6. Trail Making Test [41]: The Trails tests measure timed attention, mental flexibility and sequencing. Part A entails connecting randomly ordered numbers by drawing a line in sequence. Part B entails connecting numbers and letters in alternating order (i.e. 1, A, 2, B, etc.).
7. Digit-symbol substitution test (DSST) [37]: this test consists of nine digit-symbol pairs followed by a list of digits. Under each digit the subject should write down the corresponding symbol as fast as possible. The number of correct symbols within 90 is measured.

Multidisciplinary consensus conference: All the information obtained from the scales and the neuropsychological battery was discussed by a multidisciplinary consensus conference (in which a neuropsychologist and a physician expert in diagnosis of dementia- psychiatrist, neurologist or geriatrician, were mandatory participants) in order to ensure normal cognition, which is an inclusion criterion for the study. Normal cognition was defined as a clinical dementia rating scale (CDR) score = 0 (no dementia) and the score of the MMSE test (based on norms for age and education) corroborated by the multidisciplinary consensus conference. There were a handful of cases with gaps between the CDR and the MMSE scores. In such cases we based our decision on the CDR score which more closely reflects the effect of cognitive decline on everyday functional abilities, a decline which is required for the formal diagnosis of dementia.

### Outcome Measures

A factor analysis with varimax rotation of the comprehensive neuropsychological battery of the IDCD subjects was used to identify cognitive domains in this study. Four domains were identified. For each domain, a summary was calculated as the sum of z-scores (test scores transformed to mean zero and standard deviation one, reversed if necessary for positive values to indicate good cognition) of tests with high loadings: episodic memory (immediate and delayed recall, and recognition word list), semantic categorization [39] (letter and category fluency, and similarities), attention/working memory (diamond cancellation test, digit span forward and backward), and executive functions (Trails making A and B and the DSST). Finally, an overall cognition measure was calculated by summing the four domain summaries.

### Confounding Variables

Sociodemographic factors (age, sex, and years of education) were collected at entry into the IDCD. Cardiovascular covariates (glomerular filtration rate calculated by the MDRD formula, total cholesterol, HDL, LDL, diastolic and systolic blood pressure) were defined as means of all measurements available in the DR. Diabetes-related covariates were the estimated duration of T2D (based on time in the DR) and diabetes medications (which was categorized as oral anti-diabetic medications only, insulin only, combination of oral anti-diabetic and insulin and no medication). Since depression has been associated both with T2D [42], degree of glycemic control [43], risk for T2D- related complications [44] and with dementia [45], we also included the geriatric depression scale (GDS) score as a potential confounder. HbA1c levels at entry into the DR were defined as the third HbA1c recorded in the registry after excluding the first two HbA1c measurements, since these might reflect instability of glycemic control prior to diagnosis of T2D and treatment initiation.

### Statistical Analyses [46]

We identified distinctive trajectory groups of HbA1c using a SAS macro named PROC TRAJ [46]. This approach applies a multinomial modeling strategy to identify relatively homogenous clusters of developmental trajectories within a sample population, that is, the modeling strategy allows for the emergence of more than two trajectories. Trajectory parameters are derived by latent class analysis using maximum likelihood estimation. In particular, the distinctive trajectories of HbA1c were derived by modeling HbA1c as a function of the number of years in the DR. Quadratic curves were used to model the trends over time. The number of trajectories was determined using the guidelines suggested by Jones et al [46]. The output of PROC TRAJ includes the equations for the different trajectories along with the assignment of each patient to one of the trajectory groups. This group assignment was then used in an analysis of covariance to estimate and compare mean cognitive domain z-scores among the trajectory groups while adjusting for sociodemographic, cardiovascular, diabetes-related covariates (years in the DR and anti-diabetic medications), and GDS score.

In order to provide analyses that are comparable with those of other studies [47], we assessed the relationships of mean and standard deviations of HbA1c with cognitive function in secondary analyses, using the GLM procedure.
